# Audit of a Streamlined Multidisciplinary Team Meeting (MDT) to Results Clinic Pathway in Urology: Evaluating the Impact of a Nurse-Led Intervention on Consultant Clinic Waiting Times

**DOI:** 10.7759/cureus.97560

**Published:** 2025-11-23

**Authors:** Jonathan McAdam, Jay Atkinson, Samantha McBeigh, Hugh O'Kane

**Affiliations:** 1 Urology, Belfast Health and Social Care Trust, Belfast, GBR

**Keywords:** management pathways, nurse-led clinic, results, urologic oncology, uro oncology

## Abstract

Background: The time from uro-oncology multidisciplinary team (MDT) discussions to the outpatient results clinic was recognised as a bottleneck that could potentially delay patients' shared decision-making and timely initiation of management plans. In an effort to reduce waiting times for time sensitive patients a nurse-led results clinic was introduced to streamline select patients.

Objective: This study aimed to audit and compare waiting times from MDT discussion to consultant results clinic before and after the implementation of a nurse-led results clinic pathway.

Methods: This two-cycle audit included data from January to May 2023 and 2024 (Cycle 1, pre-intervention baseline), and prospective data from January to May 2025 (Cycle 2, post-intervention). Average waiting times across five consultant clinics were calculated on a monthly basis. A weekly eight-slot nurse-led results clinic was opened in October 2024, enabling select patients to be booked directly from MDT to have prompt discussions, shared decision-making, and timely initiation of treatment plans.

Results: The mean wait time for consultant-led clinics dropped from 34.1 days (SD 7.89) in 2023 and 2024 to 16.0 days (SD 3.16) in 2025; a 53.1% reduction. The difference was statistically significant: Welch’s t-test, t(12.75) = 6.31, p = 0.00003; paired t-test by month, t(4) = -7.64, p = 0.0016. The effect size was large (Cohen’s d = -2.66).

Conclusion: This nurse-led intervention significantly reduced waiting times for consultant-led clinics, as well as having the direct benefit of reviewing eight patients a week directly from MDT in less than a week. The model is sustainable and scalable, with implications for wider service redesign across outpatient specialties.

## Introduction

Following a urology-oncology (uro-oncology) multidisciplinary team (MDT) discussion, it is vital that we have prompt clinic discussions with patients with time-sensitive diseases to minimise anxiety, assist shared decision-making, and implement management plans as soon as possible [1]. Waiting times for consultant-led results clinics were identified as a potential bottleneck in our department’s pathway for oncology patients. These consultants have large workloads with oncology and benign services running concomitantly. This represents a systemic challenge across surgical oncology services [2]. Nurse-led clinics have demonstrated efficacy in other contexts, including prostate cancer surveillance, stoma care, and surveillance pathways [3,4]. Harnessing the expertise of nurse specialists can streamline processes, improve patient experience, and optimize consultant workload. In September 2024, our department piloted a nurse-led results clinic as an adjunct to the existing MDT-to-consultant clinic pathway. This audit aimed to evaluate the indirect effect of this intervention on waiting times for results clinic appointments across five consultant urologists in our uro-oncology service.

## Materials and methods

This was a two-cycle retrospective and prospective audit conducted within the urology department at a tertiary centre, evaluating the effect of a nurse-led results clinic on waiting times from multidisciplinary team (MDT) discussion to consultant-led results clinic. The audit cycles covered January to May 2023 and January to May 2024 (Cycle 1, pre-intervention) and January to May 2025 (Cycle 2, post-intervention). The audit was performed in a tertiary referral centre with a regional uro-oncology MDT serving a mixed population of general, oncology, and sub-specialist urology referrals. Approximately 2,100 patients are discussed annually, including around 1,400 new and 700 review cases. The study population comprised adult patients with urological malignancies discussed at the MDT whose management plans required outpatient review and counselling in a consultant results clinic.

Yearly, there are approximately 2100 patients discussed at the regional uro-oncology MDT, with 1400 being new patients and 700 being review patients. All of these cases then require a management plan to be put in place at these meetings, actioned, and part of this workload normally falls on the consultants' results clinics. To help with this a streamlined results clinic pathway was introduced in September 2024 and saw its first patients October 1st 2024. All patients were discussed at the urology MDT as per normal practice - select patients were then identified as appropriate for the nurse-led results clinic and booked directly from MDT. These cases tended to be those that require ongoing active surveillance or imaging, such as those with prostate cancer. The specialist has the ability to discuss the results with the patient, can order further investigations such as MRIs or further blood tests if needed, and play an integral role in the management of the patient's care. There is a consultant urologist cover available should they need to discuss the cases further. Prior to implementing this initiative, all patients who required further input would have been followed up by a urology consultant who was responsible for implementing the MDT decision, whether that was further investigation, onward referral, or intervention. 

Inclusion criteria consisted of adult patients with a urological malignancy who had been discussed at the regional MDT between January and May 2023, 2024, and 2025, who were booked for a consultant-led results clinic. Patients were excluded if they did not have a urological cancer or if they did not require input at a results clinic. Patients considered suitable for the nurse-led results clinic in the post-intervention period were those whose management plans had been clearly defined at MDT and did not require complex discussion. Booking for these patients was made directly by the coordinator from the MDT on the same day. A weekly nurse-led results clinic was introduced in October 2024, operating on Tuesday afternoons with eight dedicated slots. The clinic was led by an experienced Urology Clinical Nurse Specialist, who reviewed MDT outcomes with patients, discussed results, facilitated shared decision-making, and initiated treatment planning according to the consultant-approved MDT plan.

The audit included data across five consultant urologists. For each consultant, the number of days from MDT discussion to the next available results clinic was extracted monthly from the hospital’s electronic booking system. The baseline group comprised ten data points (five months each for 2023 and 2024), while the post-intervention group contained five data points (five months for 2025). Monthly averages were calculated to account for inter-consultant variation. The primary outcome parameter was the mean waiting time, measured in days, to the next available consultant results clinic. Secondary descriptive parameters included median, mode, and range of waiting times.

More than 400 patients per year can potentially be seen at this new clinic. These patients would see a reduction in waiting time for results from a mean of 31 days to 5 days (MDT Thursday, nurse-led clinic Tuesday), and the direct impact on those eight patients a week is the prompt initiation of treatment, as well as reducing the anxiety for that patient as they wait for the outcome from the MDT. An expected indirect impact of this intervention was reduced waiting times for Consultant results clinics for patients not suitable for nurse-led follow-up. Data defined as the “number of days until the next available Consultant results clinic” were harvested from clinic booking software. For five urology consultants, the monthly average waiting times for each month from January to May for each year were calculated. The datasets consisted of ten values in the baseline group (2023 and 2024) and five values in the post-intervention group (2025).

Descriptive statistics, including mean, median, mode, range, and standard deviation, were calculated for each cycle. Differences in mean waiting times between the pre- and post-intervention periods were compared using Welch’s t-test for independent samples. A paired t-test was also performed using monthly averages to account for repeated measures across consultants. Statistical significance was defined as a p-value of less than 0.05. Effect sizes were calculated using Cohen’s d for between-group comparisons and Cohen’s dz for paired comparisons. All analyses were performed using Microsoft Excel.

## Results

**Figure 1:**
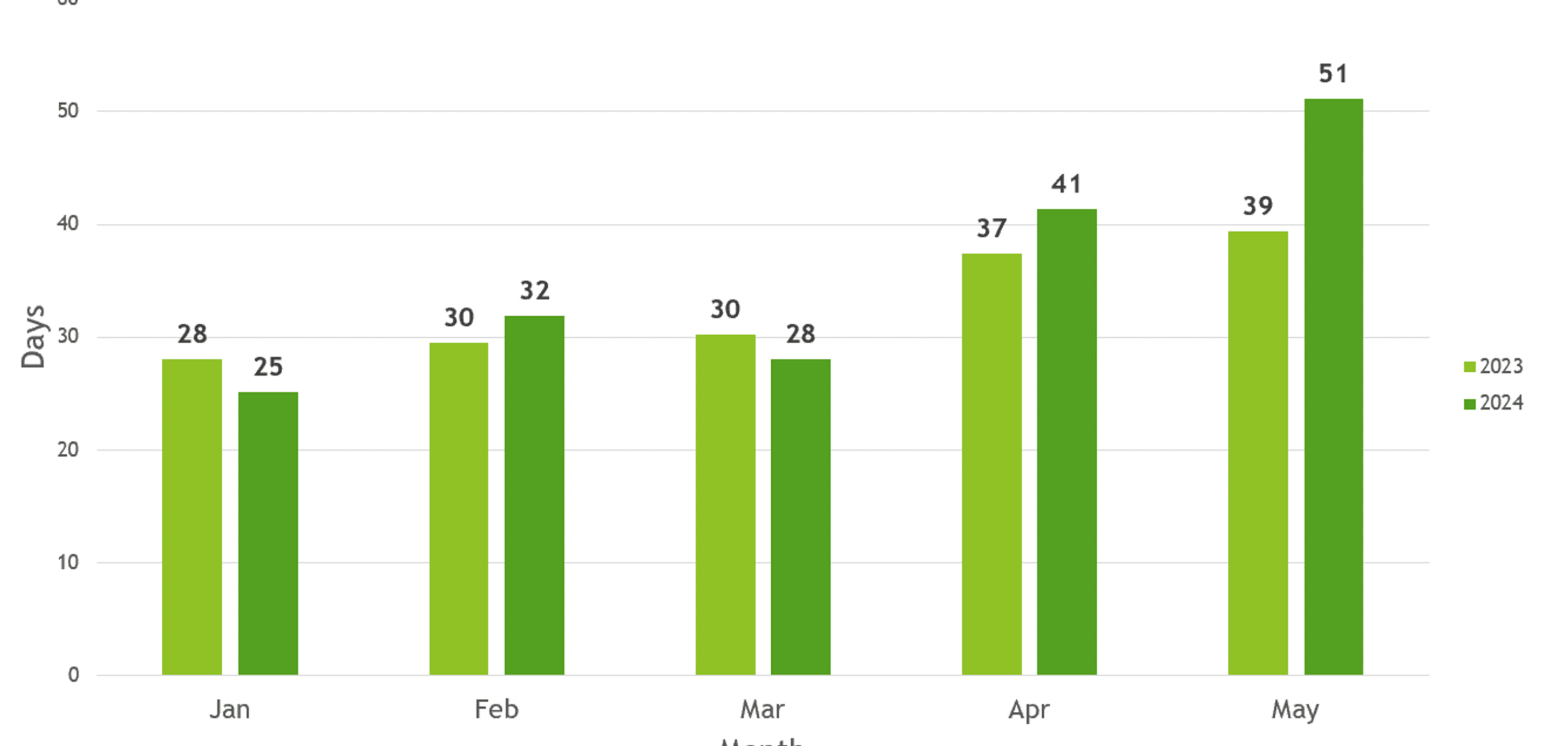
Average days to Consultant results clinic 2023/2024

**Figure 2:**
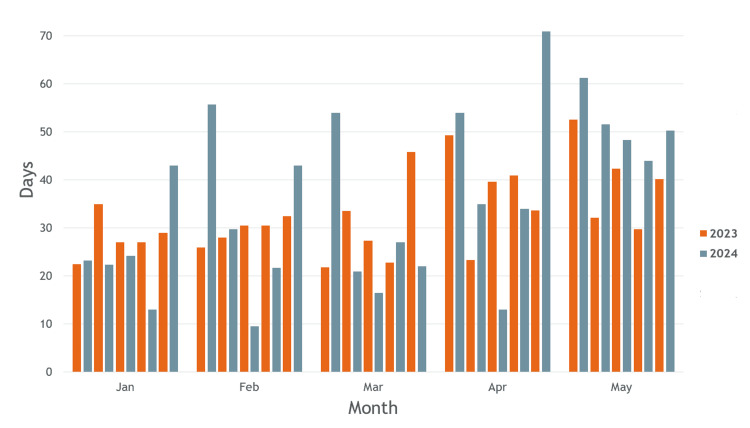
Days to Consultant results clinic 2023/2024

Following a review of the waiting time to the next available consultant, the results of the clinic appointment were collated. In the baseline period (2023-2024), the mean waiting time to the Consultant results clinic was 34.1 days with a standard deviation of 7.89 days. The average monthly waiting time for the five consultant clinics was recorded in 2023 and 2024 (Figure 1). Across the consultant cohort, the median was 31 days, and the mode was 27 days. Waiting times ranged from 10 days to 71 days (Figure 2).

**Figure 3:**
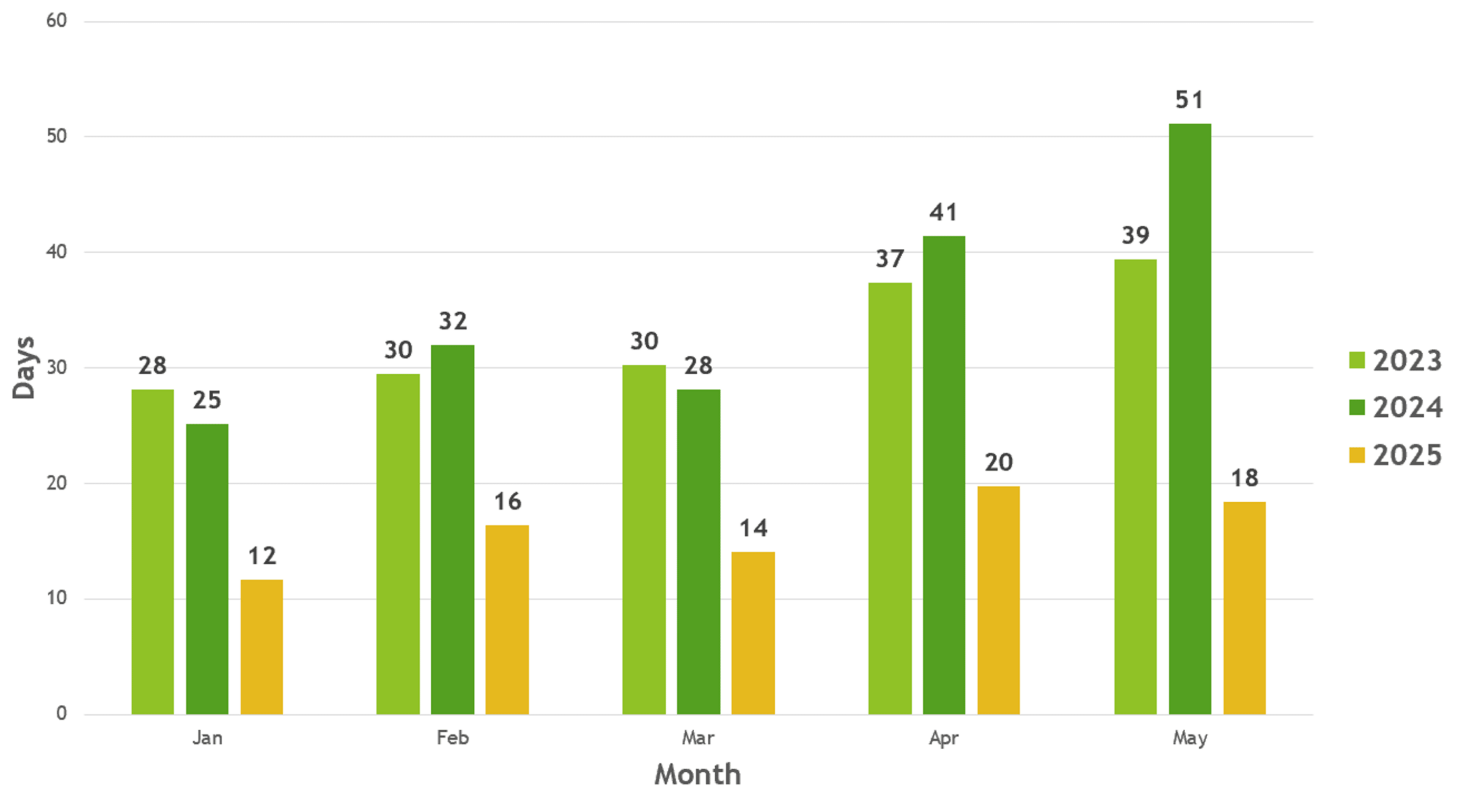
Average days to Consultant results clinic 2023/2024/2025

**Figure 4:**
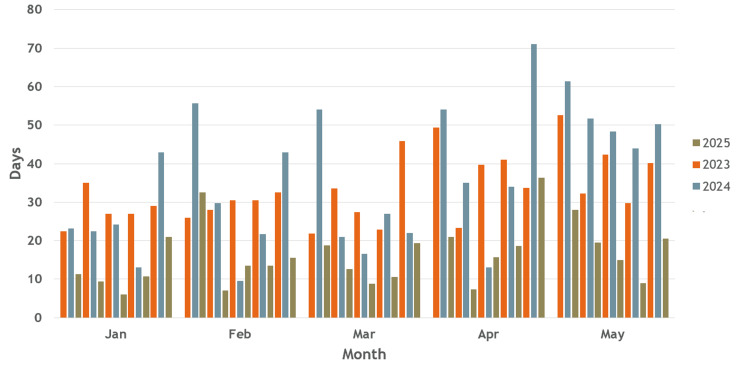
Days to Consultant results clinic

Following the implementation of the nurse-led results clinic, the waiting time to the next available Consultant results clinic was reassessed (2025); the mean waiting time was 16.0 days with a standard deviation of 3.16 days. The average for each month across the five consultants was significantly reduced (Figure 3). Across the consultant cohort, the median was 15 days, and the mode was 21 days. Waiting times until the next results clinic ranged from six days to 36 days (Figure 4).

Overall, the absolute reduction in time until the next consultant results clinic appointment was 18.1 (53.1%) days. The data was analysed using the Welch’s t-test, and the mean reduction in waiting time was statistically significant (t(12.75) = 6.31, p = 0.00003). A paired t-test by month also demonstrated that the reduction in time to consultant results clinic was significant (t(4) = -7.64, p = 0.0016). Effect sizes were large, with Cohen’s d = -2.66 and paired dz = -3.42. These results confirm a statistically significant and clinically meaningful reduction in the number of days until the next available appointment in the Consultant results clinic.

This audit demonstrated a substantial and clinically meaningful reduction in consultant results clinic waiting times following the implementation of a nurse-led results clinic. The intervention more than halved the mean waiting time, reducing it from 34 days to 16 days. This is important in the wider context of multidisciplinary cancer care, where delays in implementing MDT recommendations are strongly associated with poorer oncological outcomes and increased patient anxiety [5,6].

## Discussion

This audit demonstrated a significant improvement in waiting time for Consultant results clinic, following the introduction of a nurse-led results clinic, more than halving the mean wait from 34 days to 16 days. The direct benefit of the clinic to the more than 400 patients a year seen within a week by a nurse specialist is clear, but this audit shows the indirect benefit to all patients. This improved the consistency and speed of review in results clinics universally. The reduction in wait times and variability aligns with evidence from previous studies evaluating the role of nurse specialists in outpatient urology and oncology care. Nurse-led active surveillance in prostate cancer [7], nurse-led follow-up for lung cancer [8], and nurse-led stoma services [9] all demonstrate safety, efficiency, and patient acceptability. These services benefit from the structured, protocol-driven nature of their interventions, which aligns well with the nurse-led results clinic format adopted here. This model aligns with National Health Service (NHS) policy encouraging workforce redesign to optimise skill mix and address rising demand [10].

One of the key advantages of this model is the prompt discussion with the patient following MDT discussion, ensuring those requiring further oncology or surgical management can make informed decisions in a timely manner and are commenced on their treatment plans without unnecessary delay. By having a dedicated clinic space for select patients, we have indirectly reserved consultant appointments for less straightforward cases requiring more complicated discussion. Our intervention demonstrated the reduction in variability of waiting times, shown by the fall in standard deviation from 7.89 days to 3.16 days. Predictability of access is central to equity of care and has been emphasised in quality frameworks such as the Institute of Medicine’s six domains of healthcare quality [11].

Our findings are in keeping with other studies. For instance, Faithfull et al. evaluated nurse-led follow-up for patients undergoing pelvic radiotherapy and found equivalent clinical outcomes and patient satisfaction compared with physician follow-up, while also reducing waiting times [3]. Similarly, Moore et al. reported that nurse-led review clinics for lung cancer patients shortened follow-up intervals and enhanced continuity of care [8]. In urology, Siddharth et al. conducted a systematic review demonstrating that nurse-led cystoscopy was both safe and efficient, achieving comparable detection rates to consultant-led practice [4]. Donald et al. [12] and Kilpatrick et al. [13] likewise noted that advanced nurse practitioners integrated into oncology pathways increased accessibility and team productivity.

The magnitude of improvement in our audit, a 53% reduction in waiting times, is consistent with studies of nurse-led triage and follow-up in cancer pathways elsewhere. For instance, McCorkle et al. [14] reported that self-management and nurse-led coordination improved the timeliness of care and reduced patient-reported anxiety, while similar gains were seen in UK breast and colorectal cancer services after implementing nurse-led models [15,16]. Collectively, this evidence supports the role of nurse-led interventions as a sustainable method of addressing capacity and timeliness challenges within oncology services.

Consultant workload management was also improved. Routine results consultations were delegated to a nurse specialist, freeing consultants for urgent and complex cases. Similar redistribution of tasks has been shown to improve efficiency and outcomes in other healthcare systems [17]. The scalability of this model is promising. The digital integration of MDT outputs with booking systems could further streamline workflow. Expanding to other urology pathways (e.g., haematuria) may produce similar gains. Workforce resilience must be prioritised, with training and governance frameworks to ensure nurse specialists are adequately supported [15]. Cultural acceptance is also vital, as studies highlight that collaborative embedding of nurse-led roles leads to greater sustainability [2].

This audit has several limitations that should be acknowledged. Firstly, the post-intervention data were collected over a relatively short five-month period, which limits the ability to assess the long-term sustainability of the observed improvements. The study design was observational rather than randomised, and therefore causality between the nurse-led clinic introduction and reduction in waiting times cannot be definitively established. Other contemporaneous service changes, such as increased use of virtual consultations, staff redeployment, or catch-up effects following COVID-19-related delays, may have contributed to the observed improvements. The analysis was limited to waiting time as the primary outcome, without the inclusion of patient-reported experience, satisfaction, or clinical outcome measures, which would have provided a more holistic assessment of the intervention’s impact. Finally, this audit reflects the experience of a single tertiary centre and may not be generalisable to other institutions with differing patient populations, resources, or MDT structures.

## Conclusions

The introduction of a nurse-led results clinic within the uro-oncology MDT pathway significantly reduced waiting times for consultant-led results clinics, with mean waits decreasing from 34 to 16 days. This streamlined approach improved access, efficiency, and service capacity without increasing the workload of consultants. The model demonstrates how nurse-led interventions can optimise multidisciplinary pathways and should be maintained, monitored for long-term sustainability, and considered for adaptation across other urological and surgical specialties.
